# An *In Vitro* Model That Recapitulates the Epithelial to Mesenchymal Transition (EMT) in Human Breast Cancer

**DOI:** 10.1371/journal.pone.0017083

**Published:** 2011-02-15

**Authors:** Elad Katz, Sylvie Dubois-Marshall, Andrew H. Sims, Philippe Gautier, Helen Caldwell, Richard R. Meehan, David J. Harrison

**Affiliations:** 1 Breakthrough Breast Cancer Research Unit, Institute of Genetics and Molecular Medicine, University of Edinburgh, Edinburgh, United Kingdom; 2 Division of Pathology, Institute of Genetics and Molecular Medicine, University of Edinburgh, Edinburgh, United Kingdom; 3 MRC Human Genetics Unit, Institute of Genetics and Molecular Medicine, Edinburgh, United Kingdom; Health Canada, Canada

## Abstract

The epithelial to mesenchymal transition (EMT) is a developmental program in which epithelial cells down-regulate their cell-cell junctions, acquire spindle cell morphology and exhibit cellular motility. In human breast cancer, invasion into surrounding tissue is the first step in metastatic progression. Here, we devised an *in vitro* model using selected cell lines, which recapitulates many features of EMT as observed in human breast cancer. By comparing the gene expression profiles of claudin-low breast cancers with the experimental model, we identified a 9-gene signature characteristic of EMT. This signature was found to distinguish a series of breast cancer cell lines that have demonstrable, classical EMT hallmarks, including loss of E-cadherin protein and acquisition of N-cadherin and vimentin expression. We subsequently developed a three-dimensional model to recapitulate the process of EMT with these cell lines. The cells maintain epithelial morphology when encapsulated in a reconstituted basement membrane, but undergo spontaneous EMT and invade into surrounding collagen in the absence of exogenous cues. Collectively, this model of EMT *in vitro* reveals the behaviour of breast cancer cells beyond the basement membrane breach and recapitulates the *in vivo* context for further investigation into EMT and drugs that may interfere with it.

## Introduction

Breast cancer related deaths are primarily due to metastatic progression [1]. Understanding the mechanisms that underlie this multistep process is essential to improving clinical outcome. The transformation of normal breast epithelial cells to metastatic cancer is the result of multiple epigenetic and genetic changes, leading to deregulated interactions with the microenvironment [2]. During this process, inhibition of proliferation, cell survival, migration and differentiation is lost leading to the acquisition of an invasive phenotype. The ability to breach the basement membrane (BM) is a critical event in cancer progression and a prerequisite for metastasis. Having breached the BM, cells may then enter the lymphatic system, spread and attempt to establish themselves as distant tumor foci [3].

The trans-differentiation of cells from an epithelial to a mesenchymal phenotype is an essential part of normal embryogenesis and development [4]. Increasing evidence also supports a role for epithelial to mesenchymal transition (EMT) in the progression of many cancer types including breast, with critical roles in invasion and metastatic dissemination [5], [6]. EMT involves loss of cell-cell junctions and re-organization of the actin cytoskeleton, resulting in loss of apical-basal polarity and acquisition of a spindle-like mesenchymal morphology [7]. At the same time, there is also decreased expression of epithelial-specific proteins, including E-cadherin, which may account at least in part for the altered properties of migrating tumor cells [8], [9]. An important event in EMT is switching in expression from E-cadherin to N-cadherin [10]. In most cases this is associated with transcriptional repression of E-cadherin [9]. Several specific repressor factors have been identified including Snail, Slug, Zeb1, Zeb2 and Twist [11], all of which are zinc finger containing proteins that can bind with so called E-boxes within the *CDH1* gene promoter. N-cadherin is believed to promote cellular invasion by binding to and enhancing signalling by growth factors and is over-expressed in many invasive and metastatic human breast cancer cell lines and tumors [10], [12], [13].

Comparative analysis of mouse mammary carcinoma models and human breast tumors identified a novel human molecular subtype, termed ‘claudin-low’ cancers. These cancers are characterised by low to absent expression of genes involved in tight junctions and cell-cell adhesions, including claudins, occludins and E-cadherin [14], [15]. In addition, these moderate-high grade invasive ductal carcinomas are morphologically distinct from lobular carcinomas despite their low expression of E-cadherin [14]. Similarities between claudin-low tumors and EMT *in vitro* have been documented, however these features have not previously been compared and analysed directly. Furthermore, while the contribution of the extra-cellular matrix to the promotion of tumor progression is now appreciated [2], most current *in vitro* models do not take into account the contribution of stromal collagen into which cells undergoing EMT invade. The predisposition of tumours to undergo EMT can be enhanced by genetic alterations. For example, C35 is a 12KDa membrane-anchored protein found on the HER2 amplicon that is over-expressed in around 11% of breast cancers [16]. Cellular transformation associated with acquisition of an EMT phenotype can be induced in mammary epithelial cells transfected with a C35 expression construct resulting in increased invasion into stromal collagen, down regulation of E-cadherin and up regulation of the transcription repressor Twist [17]. This implies that collagen-invading C35-expressing cells can be used to model aspects of EMT in cancer cells.

Testing new treatments that may prevent EMT or tumor spread is challenging: conventional clinical trials may have difficulty in addressing the issues because of the ethical problems of leaving tumor *in situ*, or the limitation of a study to only very late stage disease. Robust models that can identify possible predictive biomarkers are essential. In this report, we describe a unique invasion assay, in which cell lines with known molecular pathology undergo spontaneous EMT when invading away from the basement membrane into collagen. We propose that this *in vitro* model of defined breast cancer cell lines can provide an improved representation of invasive breast cancer *in vivo,* compared to existing EMT models.

## Materials and Methods

### Gene expression analysis, RNA extraction and qRT-PCR

Microarray data was analysed using packages within Bioconductor [18] (http://www.bioconductor.org) that implement R statistical programming. Gene expression data was normalised using quantile normalisation within the BeadArray package [19] and differential gene expression assessed using Significance Analysis of Microarrays (SAM) [20] within the siggenes package. The dataset from Hershkowitz and colleagues [14] was downloaded from the UNC Microarray Database (https://genome.unc.edu/). RNA from the collagen invasion assays was labelled using a Illumina TotalPrep RNA amplification kit (Ambion) according to manufacturer's instructions. Triplicate samples from invasion assays (1500 ng cDNA per assay) were hybridised to Illumina BeadChips and whole genome gene expression analysis performed using the Illumina HumanRef-8 v3 Expression BeadChip and BeadArray Reader.

RNA from cell lines cultured on plastic was converted to cDNA prior to PCR using a QuantiTect Reverse Transcription kit (Qiagen). Gene expression patterns for invasion assays (biological triplicates) and cell lines cultured on plastic (technical triplicates) were examined using the QuantiTect SYBR Green PCR kit (Qiagen) and a Corbett RotoGene 3000. Primers for *CDH1* were: forward 5′-CGGAGAAGAGGACCAGGACT-3′, reverse 5′-GGTCAGTATCAGCCGCTTTC-3′; for *CLDN7*: forward 5′-AAAATGTACGACTCGGTGCTC-3′, reverse 5′-AGACCTGCCACGATGAAAAT; for TBP: forward 5′-GGGGAGCTGTGATGTGAAGT-3′, reverse 5′-CCAGGAAATAACTCTGGCTCA-3′; for *ACTB*: forward 5′-CCTTCCTGGGCATGGAGTCCT-3′, reverse 5′-GGAGCAATGATCTTGATCTT-3′. QuantiTect Primer Assays (Qiagen) were used for *KRT8, CRB3, MARVELD3, IRF6*, *MAL2*, *TACSTD1* and *SPINT2*. PCR program was identical for all genes: 95°C, 15 min; (94°C, 15 s; 56°C, 30 s; 72°C, 30 s)×50 cycles; 72°C, 5 min. Standard reference human cDNA was from Clontech, random primed. ∼50 ng RNA equiv/mL was used for quantification of mRNA expression. Final normalisation was performed against the geometrical mean of *ACTB* and *TBP* levels.

### Gene promoter analysis

Using the presumptive promoter region for the 9 genes (a 2 kb region upstream of the presumptive transcription start site using Ensembl 52, Jan2009, based on NCBI 36 assembly), we looked for over-represented 6- and 7-mers oligos using oligo-analysis [21] from the RSAT-tools package (http://rsat.scmbb.ulb.ac.be/rsat/) [22]. The program counts all oligonucleotide occurrences within the sequence set, and estimates their statistical significance. A calibration is done using the entire genome promoter regions as a background model (Ensembl 52, Jan2009, based on NCBI 36 assembly). For the best 7-mers candidates, we compared the obtained oligo sequences to the entire collection of consensus binding sites available in Transfac professional [23] (release 2010.1) using the compare-pattern script (RSAT-tools) and listed the associated binding factor name.

E-value for best hit 7-mer CAGGTGC/GCACCTG (2.6×10^−8^) represents the expected number of patterns which would be returned at random for a given probability. The weights in Table 1 reflect the number of matching positions, with a lower weight for matches between partially specified nucleotides (the weight for a perfect match to a 7-mer is 7). Both E-value and weights are calculated by RSAT-tools.

**Table 1:** Common transcription factor binding sites in the 9-gene signature.

Best hit	Weight	Matrix consensus	Transfac ID	Factor name
GCACCTG	6.5	ASCACCTGTTNNCA	M00044	Snail*
CAGGTGC	6.5	RACAGGTGYA	M00060	Snail*
GCACCTG	6.5	VNRCACCTGKNC	M00414	AREB6/ZEB1*
CAGGTGC	6.21	CNNCAGGTGB	M00277	LMO2 complex*
CAGGTGC	6	RRCAGGTGNCV	M00693	E12/ELSPBP1*
CAGGTGC	6	CNGNRNCAGGTGNNGNA	M00929	MyoD*
GCACCTG	5.5	YNYACCTGWVT	M00412	AREB6/ZEB1*
GCACCTG	4	RRTGNMCYTNNTGAMCCNYNT	M00966	VDR, CAR, PXR
GCACCTG	3.5	GCTGGNTNGNNCYNG	M00947	CP2/LBP-1c/LSF
GCACCTG	3.5	RGNACNNKNTGTTCT	M00957	PR/Progesterone receptor
GCACCTG	3.5	TGGCASNNNGCCAA	M01196	CTF1

A list of binding sites matching to best 7-mer found in promoters of the common EMT gene signature. Three muscle initiator sequences with no further information were excluded.

*E-box binding transcription factors. E12 is part of the LMO2 complex.

### Cell lines

MCF10A, Hs578T, HBL100, BT549, MDA-MB157, MDA-MB231 and MDA-MB436 cell lines were obtained from American Type Culture Collection. SUM159PT and SUM1315MO2 cells were a kind gift from Akira Orimo (University of Manchester). The cells were cultured as previous described [24] at 37 deg C, 5% CO2: MCF10A in DMEM/F12 media (Invitrogen) with 5% horse serum (Invitrogen), 20 ng/ml EGF, 100 ng/ml cholera toxin, 0.01 mg/ml insulin and 500 ng/ml hydrocortisone (all from Sigma); MDA-MB157, MDA-MB231, HBL100 and HS578T in DMEM, 10% bovine serum (both from Invitrogen); SUM159PT in Ham's F12 (Invitrogen), 5% bovine serum, insulin, hydrocortisone; MDA-MB436 in L15 (Invitrogen), 10% bovine serum; BT549: RPMI-1640, 10% bovine serum; SUM1315MO2 in Ham's F12, 5% bovine serum, insulin, EGF.

### Primary cell isolation for tissue culture

Fresh normal breast tissue and breast tumor materials were incubated for 1 hour at room temperature in tissue mix consisting of DMEM/F12, 1% fungizone, 1000 U/ml penicillin, 1000 µg/ml streptomycin, 10 µg/ml insulin and 10% bovine serum (all from Invitrogen). Tissue cores were then finely chopped (∼1 ml pieces) and put in a tissue mix/Collagenase I solution (Invitrogen; made up with 200 µL of 200 U/ml Collagenase I to 20 ml tissue mix) for digestion (2 hours at 37 deg C, 200 rpm). The digested tissue was then spun for 4 mins at 60 g. The resulting pellet was plated with fibroblast media (DMEM supplemented with 10% bovine serum, 50 U/ml penicillin and 50 mg/ml streptomycin) and the supernatant spun for a further 4 mins at 600 g, 4 times. The resulting second pellet (mammary epithelial cells) was plated with HMEC media (CnT-22 (Cellntec) supplemented with 5% FCS).

### Ethics Statement

The use of primary breast cells was approved by the Lothian Research Ethics Committee (08/S1101/41). Materials were obtained with written informed consent from all participants involved in this study.

Rat tails obtained from animals at the University of Edinburgh animal facilities scarified for other scientific purposes and did not require ethical approval.

### SDS-PAGE

Protein lysates (50 µg/well, as determined by MicroBCA protein assay) were resolved by SDS-PAGE after being denatured for 1 hour at 60 deg C. The resolving gel (7.5% w/v acrylamide, 0.37 M TRIS pH 8.85, 0.1% SDS, 0.02% AMPS, 0.25% TEMED; all from Sigma) was set between glass plates using a Bio-Rad kit. Once the resolving gel had set, a stacking gel (3.6% w/v acrylamide, 0.12 M TRIS pH 6.8, 0.1% SDS, 0.03% AMPS, 0.33% TEMED) was layered and a comb used to create wells for sample loading. The loaded samples were electro-separated under constant current (100–200 mA) using electrophoresis buffer (25 mM Trizma Base, 0.19 M Glycine, 10% SDS). Electro-transfer onto immobilon transfer membrane (Millipore) was performed using transfer buffer (25 mM Trizma Base, 0.19 M Glycine) using a Bio-Rad kit, under constant electrical potential (∼30 mV for at least 2 hours).

### Western Blotting

Nonspecific binding was blocked with Li-Cor Odyssey Blocking Buffer (Li-Cor), diluted 50∶50 in PBS, for 1 hour at room temperature. Primary antibodies were diluted in Li-Cor Odyssey Blocking Buffer, diluted 50∶50 in 0.1% PBS-Tween20, and incubated with the blot overnight at 4 deg C. Blots were washed 3 times for 5 mins with PBS-T before incubation with appropriate fluorescent secondary antibodies (Li-Cor), diluted 1∶10,000 in Li-Cor Odyssey Blocking Buffer, diluted 50∶50 in 0.1% PBS-Tween20, for 45 mins at room temperature. Exposure to light was avoided. Subsequently, membranes were washed, dried and scanned on the Li-Cor Odyssey scanner. All washes/incubations were carried out under constant agitation. Primary antibodies used as follows: E-cadherin, BD, 610181, Mouse, 1∶2500; Claudin7, Abcam, Ab75347, Rabbit, 1∶1000; N-cadherin, BD, 610921, Mouse, 1∶3000; Vimentin, Sigma, V 6630, Mouse, 1∶1000; Zeb2, BD, 611256, Mouse, 1∶250; Slug, LifeSpan Bio, LS-C30318, Rabbit, 1∶4000; Snail, Abcam, ab17732, Rabbit, 1∶4000; Tubulin, Abcam, Ab7291, Mouse, 1∶6000.

### Rat tail collagen I preparation

Fresh rat tails were collected and frozen. Prior to harvesting these were placed in 70% ethanol. Tendons were stripped from the tails and returned to 70% ethanol to sterilise. The collected tendons were weighed and transferred to the appropriate volume of pre-cooled acetic acid (1 g tendon to 250 ml 0.5 M acetic acid) and gently stirred for 48 hours at 4 deg C. The tendon/acetic acid mix was then centrifuged at 10,000 g for 30 mins and the pellet discarded. An equal volume of 10% (w/v) NaCl was added to the supernatant and the mix allowed to stand overnight at 4 deg C. The collagen-rich, insoluble ‘bottom layer’ was taken and collected by further centrifugation (10,000 g for 30 mins). The collagen-rich material was resuspended in 0.25 M acetic acid at 4 deg C and dialysed against 1∶1000 acetic acid at 4 deg C for 3 days, changing the dialysis buffer twice daily. The collagen solution was then sterilised by centrifugation (20,000 g for 2 hours) and stored at 4 deg C. Collagen was diluted as required by the addition of sterile 1∶1000 acetic acid to a stock concentration of 1.2 mg/ml.

### Establishment of 3D invasion assays

200 µL cell-collagen plugs and 75 µL cell-Matrigel plugs were made in a u-shaped 96 well plate, with the aim of achieving comparable size after a 24 hr incubation (day -1). A cell concentration of 1×10^6^ was used for all plugs. Rat tail collagen I, for both plugs and subsequent embedding, was prepared as per the ‘on top’ assays. Growth factor reduced Matrigel was obtained from BD and used at a 5 mg/ml. Matrigel matrix is a soluble basement membrane extract of the Engelbreth-Holm-Swarm tumor that gels at room temperature to form a reconstituted basement membrane. The major components are laminin, collagen IV, entactin and heparin sulphate membrane. After the 24 hr incubation, cell plugs were carefully removed from their 96 well plate and embedded in 1 ml of collagen in a 24 well plate (taken as day 0), with or without fibroblasts (used at 10,000/ml). These cultures were incubated for a further hour and then carefully freed from the edges of the well (to allow contraction of the collagen) and supplemented with 0.5 ml of cell-specific media. The cultures were then left to invade. Media was changed weekly. Gels were fixed at either 1 or 2 weeks in 10% phosphate buffered formalin and wax embedded.

### Immunofluorescence

Immunofluorescence was preformed as described previously [17]. Briefly, antigen retrieval for all epitopes was carried out using heat treatment under pressure in a microwave oven for 5 min in citrate buffer (82 ml 0.01 M sodium citrate: 18 ml 0.01 M citric acid) pH 6.0. Slides were incubated with primary antibodies for 1 hr at room temperature. Primary antibodies were as follows: E-cadherin, BD, 610181, Mouse, 1∶1500; N-cadherin, BD, 610921, Mouse, 1∶300; Zeb2, BD, 611256, Mouse, 1∶50; Slug, LifeSpan Bio, LS-C30318, Rabbit, 1∶1000; Snail, Abcam, ab17732, Rabbit, 1∶700. For Snail staining, mouse anti-pancytokeratin (Invitrogen, 1∶25) was added to visualise epithelial cells. Mouse primary antibodies were incubated overnight with rabbit anti-pancytokeratin (Dako, 1∶150). The epithelial compartment was then visualised with Cy3 (Invitrogen, anti-rabbit; anti-mouse, both used at 1∶25). DAPI (4′,6-diamidino-2-phenylindole) counterstain (Invitrogen) was used to identify nuclei and Cy-5-tyramide (HistoRx, 1∶50) was used to detect protein ‘targets’. Monochromatic images of each TMA core were captured at 20× objective using an Olympus AX-51 epifluorescence microscope, and high-resolution digital images were analyzed by the AQUAnalysis software [17].

## Results

### Identification of a common EMT signature in the breast

**Figure 1:**
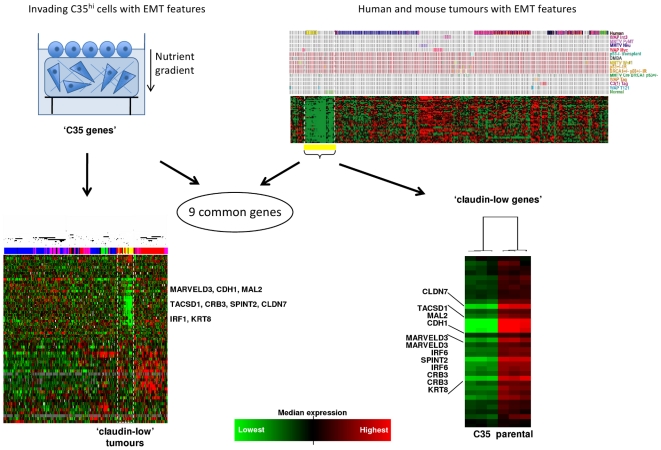
Comparison of genes correlating with C35 expression and those identifying the claudin-low phenotype identifies a 9-gene EMT signature. The 100 illumina probes most significantly differentially expressed between collagen-invading C35 and parental cells were represented by 57 genes that were able to cluster together the 13 claudin-low tumors identified by Herschkowitz and colleagues (*left panels*). A set of 34 claudin-low genes from the Herschkowitz were all significantly down-regulated in C35-expressing cells compared to parental cells (*right panels*). A signature of nine EMT-related genes is shared between the C35 and claudin-low gene lists (full lists in Fig. S1).

In order to establish an *in vitro* EMT signature, we identified a set of 57 genes that strongly correlated with C35-induced EMT *in vitro* using significance analysis of microarrays (SAM, [20]). These ‘C35 genes’ were subsequently found to be sufficient to cluster claudin-low tumors together in a breast cancer dataset [14] (Figs. 1 and S1). In addition, a 34 gene ‘claudin-low’ signature identified in murine mammary carcinoma and human breast tumors [14], was significantly down-regulated in collagen-invading C35-expressing cells in comparison to parental cells (range p = 0.048 to p = 1×10^−8^; Figs. 1 and S1). Nine genes were common between the ‘C35 genes’ and ‘claudin-low genes’ signatures (Fig. 1): *CDH1*, *CLDN7*, *CRB3*, *KRT8*, *TACSTD1*, *IRF6*, *SPINT2, MAL2* and *MARVELD3*. Five of these, *CDH1* (E-cadherin), *CLDN7* (Claudin-7), *TACSTD1* (EpCAM), *IRF6* and *KRT8* (Keratin-8) have been previously implicated by their low expression in claudin-low cancers and/or in EMT *in vitro* [25], [26], [27]. *SPINT2* (Hepatocyte growth factor activation inhibitor-2, HAI-2) is capable of regulating a HGF-induced invasion of human breast cancer cells [28]. Two novel genes found to be down-regulated: the apical sorting protein *MAL2* [29] and its tight-junction-associated homologue *MARVELD3* [30].

We determined whether the nine EMT genes share common regulatory elements in their promoters and identified a shared 7-mer: CAGGTGC/GCACCTG. This binding motif is targeted by E-box transcription repressors, including Snail and ZEB families (Table 1) raising the possibility that these transcription factors repress all nine genes in the EMT pathway both *in vitro* and *in vivo*.

### Identification of cell lines with ‘claudin-low’ features

**Figure 2:**
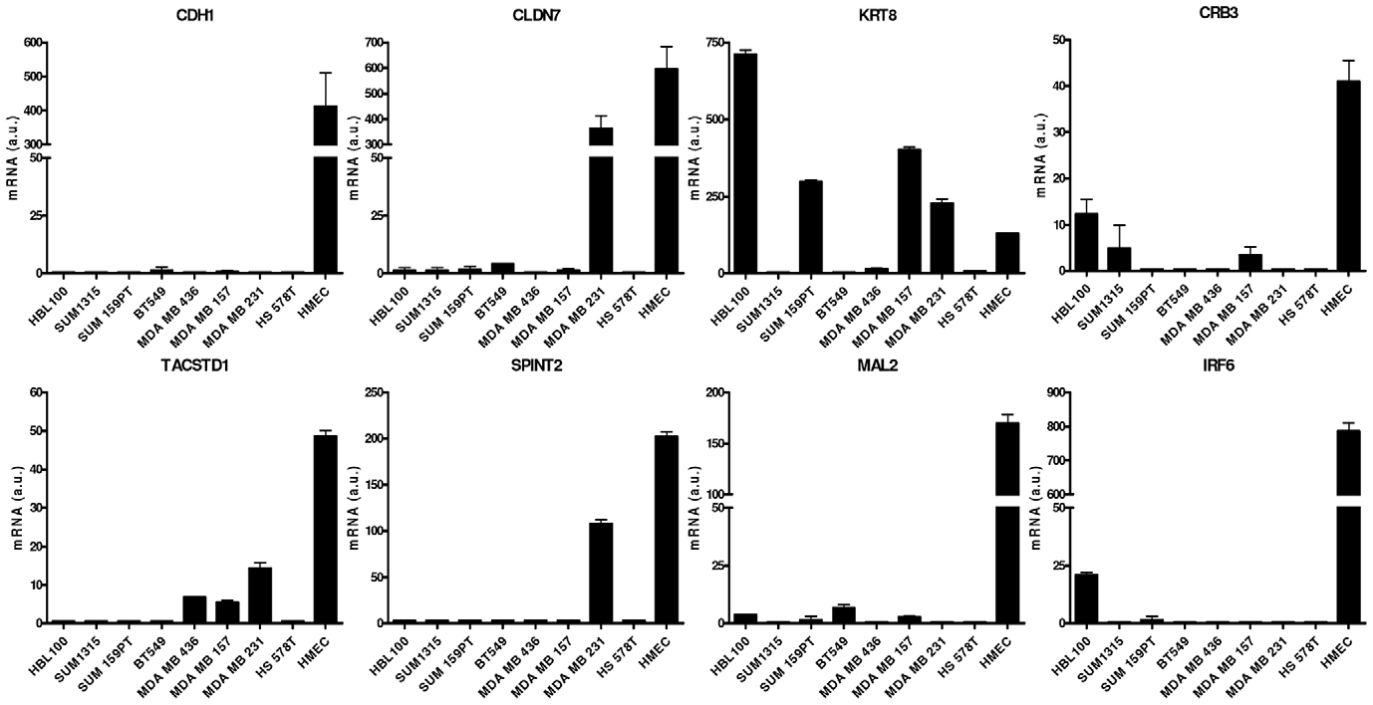
The 9-gene C35/claudin-low signature is down-regulated in a subset of human breast cell lines. Eight cell lines exhibit low expression of *CDH1*, *CLDN7*, *CRB3*, *KRT8*, *TACSTD1*, *IRF6*, *SPINT2* and *MAL2* when cultured on plastic. *MARVELD3* could not be assessed due to particularly low levels of expression. Technical triplicate mRNA expression data is shown for each line. HMEC cDNA is shown for comparison.

The 9-gene signature was identified in nine breast cancer cell lines from a previously published gene expression dataset [24] that all expressed low levels of the EMT genes: cell lines BT549, Hs578T, HBL100, MDA-MB157, MDA-MB231, MDA-MB435, MDA-MB436, SUM1315MO2 and SUM159PT respectively. We excluded the MDA-MB435 line from this cohort of cell lines due to doubts as to its tissue of origin [31]. The remaining eight cell lines show clear mesenchymal morphology when cultured on plastic (Fig. S2). We confirmed down-regulation of eight of the nine EMT genes by quantitative RT-PCR (Fig. 2) using normal human mammary epithelial cells (HMECs) as a positive control. We also validated low expression of these genes in the C35 model ([17] and data not shown).

**Figure 3:**
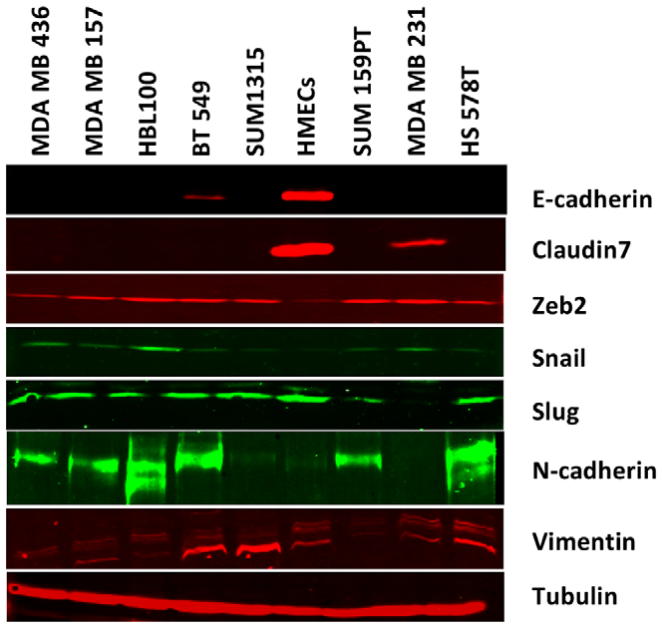
Claudin-low-like cell lines express key markers of EMT. Western blots demonstrate the expression of EMT-related markers at the protein level. HMEC lysates are shown for comparison.

Western blotting was used to investigate the expression patterns of EMT-related proteins, including transcription repressors. All lines exhibit low levels of E-cadherin and Claudin-7 in comparison to normal mammary epithelial cells (Fig. 3), whereas ZEB2 (SIP-1), an E-box transcription factor that can induce EMT, is expressed in all the cell lines with claudin-low features. Most of the cell lines also have detectable expression of Snail, whereas Slug is absent in only one (MDA-MB231). Lastly, all of the cell lines express the mesenchymal marker vimentin and seven of the cell lines have detectable expression of N-cadherin.

### A 3D invasion assay that mimics invasion into stromal collagen

**Figure 4:**
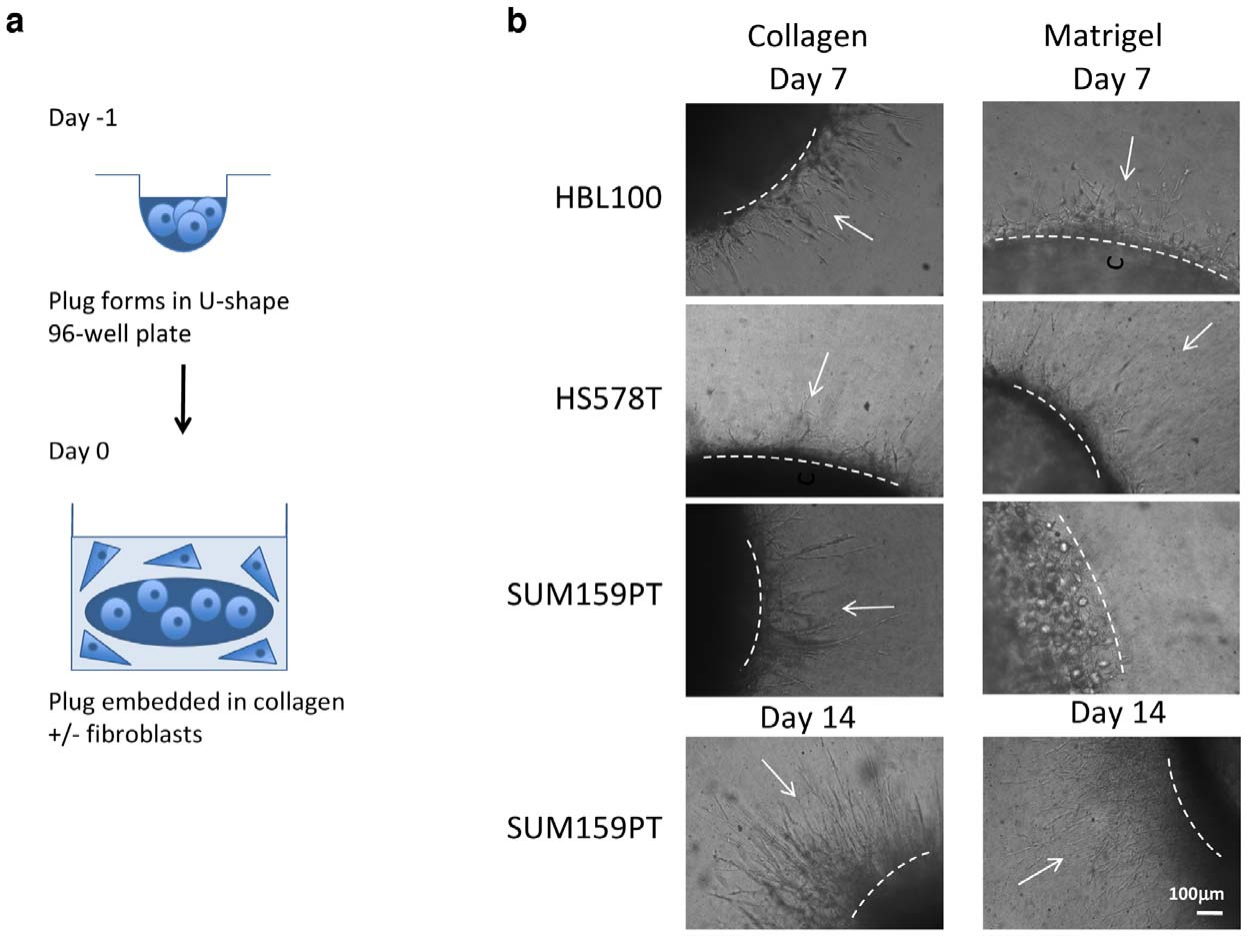
A novel invasion assay mimics EMT. (a) Schematic illustration of the ‘plug’ invasion assays. A collagen- or Matrigel-based epithelial plug is embedded in additional collagen, with or without fibroblasts. Epithelial cells then invade in a star-burst manner into surrounding collagen. (b) Morphological changes suggestive of spontaneous transitions between MET and EMT states are observed by light microscopy. Cell-collagen plugs were made with HBL100, HS578T and SUM159PT cell lines. These exhibit a predominantly elongated morphology at day 0. Clear invasion into surrounding collagen is seen by day 7 (*left panels*, arrows). Cell-Matrigel plugs with the same lines exhibit a rounded morphology on day 0. By day 7, HBL100 and HS578T cells have reverted to an elongated morphology and are invading into surrounding collagen. In contrast, SUM159PT cells retain a rounded morphology accompanied by delayed invasion (day 7) although this appears to be overcome by day 14 (*right panels*). Dotted lines represent the original plug edge. Bar = 100 µm.

A critical event in cancer progression is the acquisition of an invasive phenotype, and in particular the ability to breach the basement membrane (BM) into the stromal collagen. We developed a 3D model that attempts to mimic this process. Histologically normal breast epithelial cells are first embedded in a laminin-rich, BM-like Matrigel to generate a cell ‘plug’ which was subsequently embedded in collagen to mimic the surrounding extracellular matrix (Fig. 4a). This model potentially generates a three-stage assay that allows investigation of cells: i) contained by BM; ii) as they invade across BM; iii) as they invade more distally into surrounding collagen. In addition, the movement of cells in a horizontal plane can easily be followed by light microscopy, in contrast to the movement of cells in a vertical plane that occurs with the collagen-based ‘on top’ assay [17].

**Figure 5:**
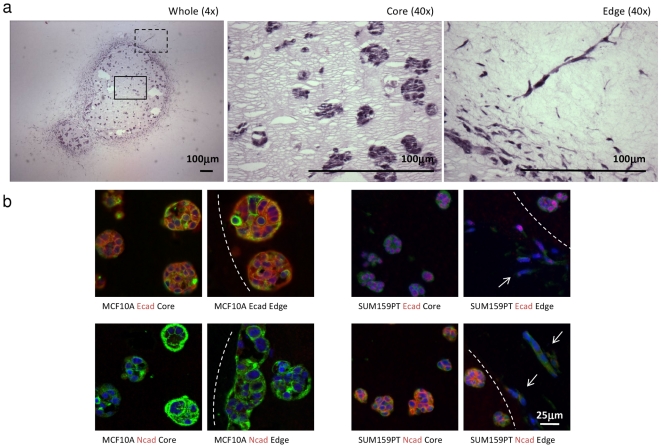
Changes in cells undergoing EMT while invading collagen stroma *in vitro*. (a) SUM159PT cell-Matrigel plugs were fixed at day 14 to monitor morphological changes during collagen invasion. Images of the whole plugs (4× magnification, *left panel*), core (*middle panel*) and plug edge (*right panel*) are shown (both 40× magnification). Note the organised, rounded morphology in Matrigel (*middle panel*) in contrast to the elongated morphology as cells invade into surronding collagen (*right panel*), indicative of EMT. (b) E-cadherin expression in MCF10A cells is comparable to N-cadherin expression in SUM159PT cells. Representative immunofluorescence images of E-cadherin and N-cadherin protein expression in MCF10A (*left panel*) and SUM159PT cells (*right panel*) are shown. Expression within the plug (core) and at the edge where cells are seen to invade surrounding collagen (arrows) is compared. Note the change in morphology as cells invade. Bar = 50 µm.

Three different cell lines with low expression of the 9-gene EMT signature (HBL100, HS578T and SUM 159PT) demonstrated clear and reproducible invasion in this novel assay. Importantly, all three cell lines adopt a round morphology when embedded in Matrigel (day 0), versus the predominantly elongated morphology that is seen in collagen (Fig. S3). By day 7, the HBL100 and HS578T cells have reverted to an elongated morphology, indistinguishable from that seen in collagen, and are invading across BM and into surrounding collagen. In contrast, many SUM159PT cells retain a round morphology, accompanied by delayed invasion (Fig. 4b). By day 14, SUM159PT cells appear to have overcome this inhibition and many elongated cells are now seen leaving the Matrigel plug. Those cells that remain in the Matrigel plug still retain a more round morphology (Fig. 5a). MCF10A cells (a non-transformed line) were also tested in this assay and do exhibit an invasion phenotype. As expected, MCF10A cells appear to form polarised, growth arrested structures [32]. These observations suggest that this model may allow the investigation of cells as they invade across the BM. Importantly, SUM159PT cells are the most affected by Matrigel in terms of morphology and invasive capacity, and were therefore selected for further investigation.

SUM159PT cells, which are an excellent metastasis model *in vivo* [33], [34], were selected for further EMT analysis with MCF10A cells serving as a control, as they show uniform, membranous expression of E-cadherin and no expression of N-cadherin. In contrast, SUM159PT cells show no membrane-specific E-cadherin expression but do show membranous N-cadherin expression throughout the core of the plug (Fig. 5b). In the elongated invading cells at the periphery N-cadherin expression appears to be down-regulated.

Stromal fibroblasts have been shown to play critical roles in some models of invasion, remodelling the ECM and generating tracks along which epithelial cells can follow [35]. The role of normal and cancer-associated fibroblasts (CAFs) was therefore also investigated. No difference in invasion was evident with both normal fibroblasts and CAFs. This lack of effect on invasion was seen when epithelial cells were embedded in both collagen and Matrigel (Fig. S4).

## Discussion

This study identifies 9 key genes shared by breast cells undergoing EMT *in vitro* and EMT enriched claudin-low tumors. This signature in turn was used to identify breast cancer cell lines that are potentially useful in studying EMT *in vitro*. A 3D invasion model was developed that specifically addresses the link between EMT and invasion into stromal collagen in these cell lines, which may be representative of a general behaviour. This novel model was used to examine the expression patterns of cadherins in the EMT cell lines when invading from the basement membrane context to a collagen-rich environment.

The association of claudin-low breast cancer and epithelial to mesenchymal transition is now well established [14], [27], [36] and cell lines can be identified with gene expression profiles similar to those of claudin-low tumors [27]. The low expression signature is also found in these Basal B/mesenchymal/claudin-low cell lines, identified elsewhere [27]. Importantly, our results do not single out a particular EMT inducing transcriptional repressor, although these are broadly expressed (ZEB2, Snail and Slug) in the cell lines. This suggests that the induction of EMT may result from a combination of factors, resulting in repression of common downstream molecules. From a functional point of view, this is consistent with loss of cell-cell contact as a prerequisite for the detachment of invading cells from the tumor mass and their penetration of surrounding stroma [37].

Previously published invasion models have used either pure collagen environment [38] or non-physiological methylcellulose [39]. More physiologically relevant basement membrane-containing models, such as the chick chorioallantoic membrane [40] or peritoneal basement membrane [41], are inflexible, difficult to scale up and often have a very low yield. Our *in vitro* invasion model potentially offers a deeper investigation of the nature of EMT. The combination of basement membrane environment and surrounding collagen stroma maintains and mimics aspects of EMT *in vivo*.

The 3D model demonstrated here exemplifies how using the same cell line simultaneously in both basement membrane environment and in tissue-like collagen matrix may enable a better understating of EMT. Two novel observations were made using this model: within the basement membrane plug, N-cadherin expression in cells with EMT signature can phenocopy E-cadherin expression in normal mammary epithelial cells, maintaining a tight round morphology; and surprisingly, N-cadherin is lost as cells with EMT signature invade.

Claudin-low breast cancers are likely to represent the most acute EMT phenotype *in vivo*, but other subtypes may also present some EMT features [15], [42]. The current study has extended our understanding of common mechanisms of EMT in breast cancer. This study showed that the down-regulation of cell-cell contact molecules in claudin-low cancers is accompanied by changes in HGF signalling and apical sorting molecules. Furthermore, the 3D model has questioned the concept of a ‘cadherin switch’ *in vivo*. We have also observed elsewhere that in invasive ductal breast carcinomas there is no inverse correlation between E-cadherin and N-cadherin protein expression levels (S. Dubois-Marshall and E. Katz, unpublished observations). This raises the possibility that single cell invasion is cadherin-independent. This will to be verified in future experiments examining other cadherin molecules involved in cell motility, such as cadherin-11 [13]. Taken together, the 3D model presented here gives an opportunity to explore these possibilities relating to EMT as it may occur *in vivo* in claudin-low breast cancers and beyond.

## Supporting Information

Figure S1 **Comparison of genes correlating with C35 expression and those identifying the claudin-low phenotype.** Full details of C35 and claudin-low signatures shown in Fig. 1.
(TIF)

Figure S2 **Claudin-low cell lines exhibit a mesenchymal morphology.** Eight claudin-low cell lines were identified. Representative live microscopy images of these lines cultured on plastic are shown. The non-transformed cell line, MCF10A, is shown for comparison. Bar = 100 µm.
(TIF)

Figure S3 **Morphology of cell-collagen assays.** SUM159PT cell-collagen plugs were fixed at day 14 following a period of invasion Images of the whole plugs (4× magnification, *left panel*), core (*middle panel*) and plug edge (*right panel*) are shown (both 40× magnification). Note the consistently elongated cell morphology unlike cell-Matrigel assays (Figure 5a).
(TIF)

Figure S4 **Comparable invasion of SUM159PT cells regardless of the presence or type of fibroblasts in the surrounding collagen.** Comparable invasion of SUM159PT cells is seen with no, normal and cancer-associated fibroblasts. This is seen with both cell-collagen (*top panel*) and cell-Matrigel (*bottom panel*) plugs. H&E staining relating to fixation at day 6 is shown here. Bar = 100 µm.
(TIF)
