# Correction: An *In Vitro* Model That Recapitulates the Epithelial to Mesenchymal Transition (EMT) in Human Breast Cancer

**DOI:** 10.1371/annotation/c605af1d-7e20-483a-bee0-eba724cc3c19

**Published:** 2011-04-05

**Authors:** Elad Katz, Sylvie Dubois-Marshall, Andrew H. Sims, Philippe Gautier, Helen Caldwell, Richard R. Meehan, David J. Harrison

There is an error in sentence seven of the "Western Blotting" section of Materials and Methods. The sentence should read: "Primary antibodies used as follows: E-cadherin, BD, 610181, Mouse, 1:2500; Claudin7, Abcam, Ab75347, Rabbit, 1:1000; N-cadherin, BD, 610921, Mouse, 1:3000; Vimentin, Sigma, V 6630, Mouse, 1:1000; Slug, LifeSpan Bio, LS-C30318, Rabbit, 1:4000; Snail, Abcam, ab17732, Rabbit, 1:4000; Tubulin, Abcam, Ab7291, Mouse, 1:6000."

