# Correction: An *In Vitro* Model That Recapitulates the Epithelial to Mesenchymal Transition (EMT) in Human Breast Cancer

**DOI:** 10.1371/annotation/a16ffa9e-6281-44f0-98ac-9e6302987fdb

**Published:** 2011-04-05

**Authors:** Elad Katz, Sylvie Dubois-Marshall, Andrew H. Sims, Philippe Gautier, Helen Caldwell, Richard R. Meehan, David J. Harrison

There is an error in paragraph two, sentence two of the "Identification of cell lines with 'claudin-low' features" section of Results. The sentence should read: "All lines exhibit low levels of E-cadherin and Claudin-7 in comparison to normal mammary epithelial cells (Fig. 3)."

