# Correction: An *In Vitro* Model That Recapitulates the Epithelial to Mesenchymal Transition (EMT) in Human Breast Cancer

**DOI:** 10.1371/annotation/3853c13c-6fb5-48a5-8a86-13353647edd4

**Published:** 2011-04-05

**Authors:** Elad Katz, Sylvie Dubois-Marshall, Andrew H. Sims, Philippe Gautier, Helen Caldwell, Richard R. Meehan, David J. Harrison

There is an error in sentence four of the "Immunofluorescence" section of Materials and Methods. The sentence should read: "Primary antibodies were as follows: E-cadherin, BD, 610181, Mouse, 1:1500; N-cadherin, BD, 610921, Mouse, 1:300; Slug, LifeSpan Bio, LS-C30318, Rabbit, 1:1000; Snail, Abcam, ab17732, Rabbit, 1:700."

