# Correction: An *In Vitro* Model That Recapitulates the Epithelial to Mesenchymal Transition (EMT) in Human Breast Cancer

**DOI:** 10.1371/annotation/3aa3ef2b-b38e-4f37-ad5d-c1448d722b26

**Published:** 2011-04-05

**Authors:** Elad Katz, Sylvie Dubois-Marshall, Andrew H. Sims, Philippe Gautier, Helen Caldwell, Richard R. Meehan, David J. Harrison

There is an error in paragraph two, sentence three of the Discussion. The sentence should read: "Importantly, our results do not single out a particular EMT inducing transcriptional repressor, although these are broadly expressed (Snail or Slug) in the cell lines."

