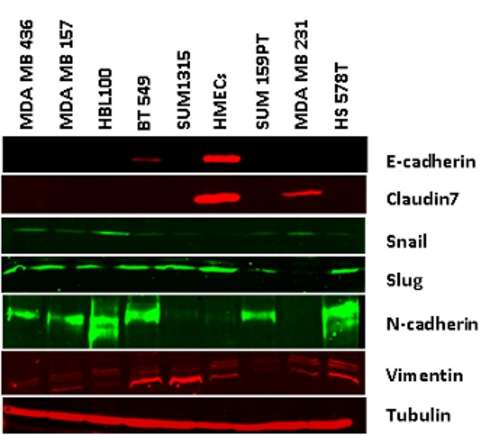# Correction: An *In Vitro* Model That Recapitulates the Epithelial to Mesenchymal Transition (EMT) in Human Breast Cancer

**DOI:** 10.1371/annotation/b9ee2908-d86b-4e21-86b7-4d0151ad538a

**Published:** 2011-04-06

**Authors:** Elad Katz, Sylvie Dubois-Marshall, Andrew H. Sims, Philippe Gautier, Helen Caldwell, Richard R. Meehan, David J. Harrison

The antibody "Zeb2" should not have appeared in Figure 3. Please view the correct Figure 3 here: 

**Figure:**