# Supplementary figures and images for: An In Vitro Model That Recapitulates the Epithelial to Mesenchymal Transition (EMT) in Human Breast Cancer
Source: PLoS One. 2011 Feb 15;6(2):e17083. doi: 10.1371/journal.pone.0017083 (PMC3039655; doi:10.1371/journal.pone.0017083)

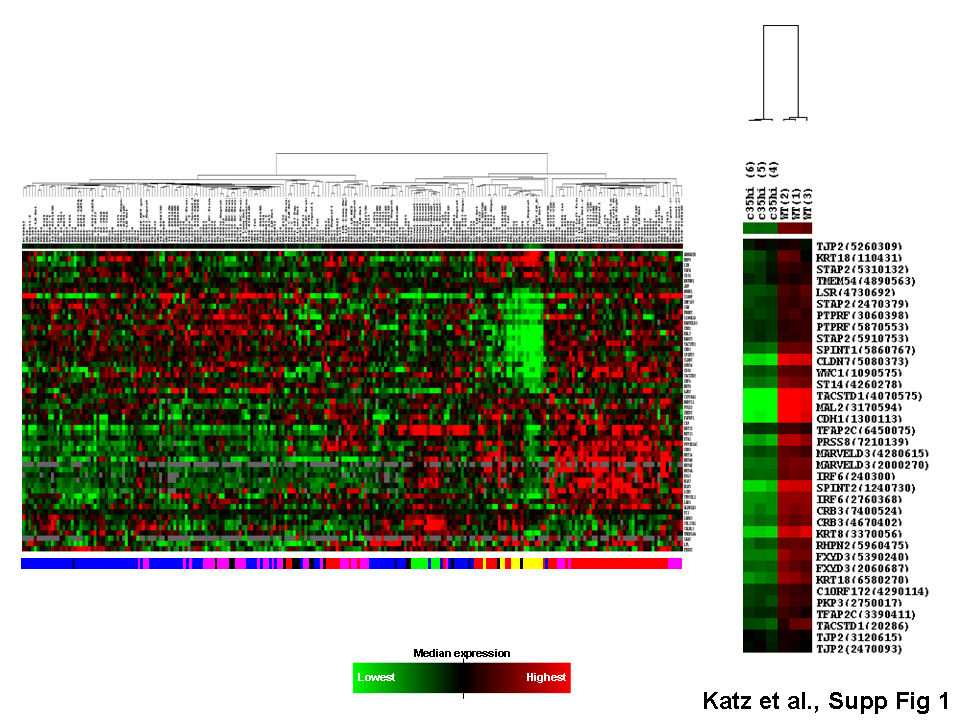

Supplement: Figure S1 — Comparison of genes correlating with C35 expression and those identifying the claudin-low phenotype. Full details of C35 and claudin-low signatures shown in Fig. 1. (TIF) [file pone.0017083.s001.tif]

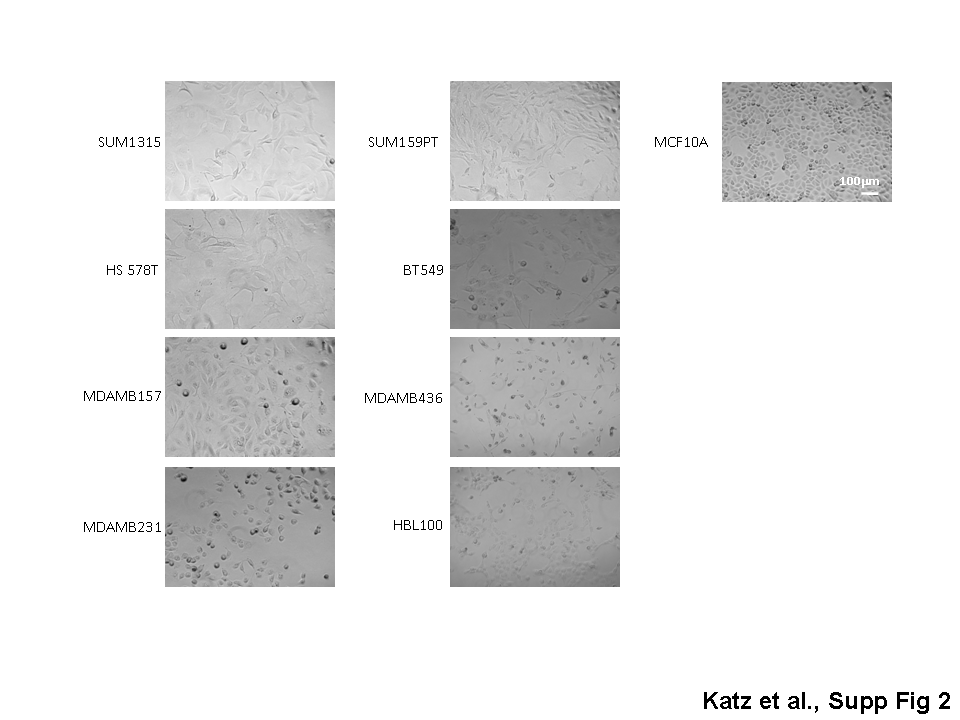

Supplement: Figure S2 — Claudin-low cell lines exhibit a mesenchymal morphology. Eight claudin-low cell lines were identified. Representative live microscopy images of these lines cultured on plastic are shown. The non-transformed cell line, MCF10A, is shown for comparison. Bar = 100 µm. (TIF) [file pone.0017083.s002.tif]

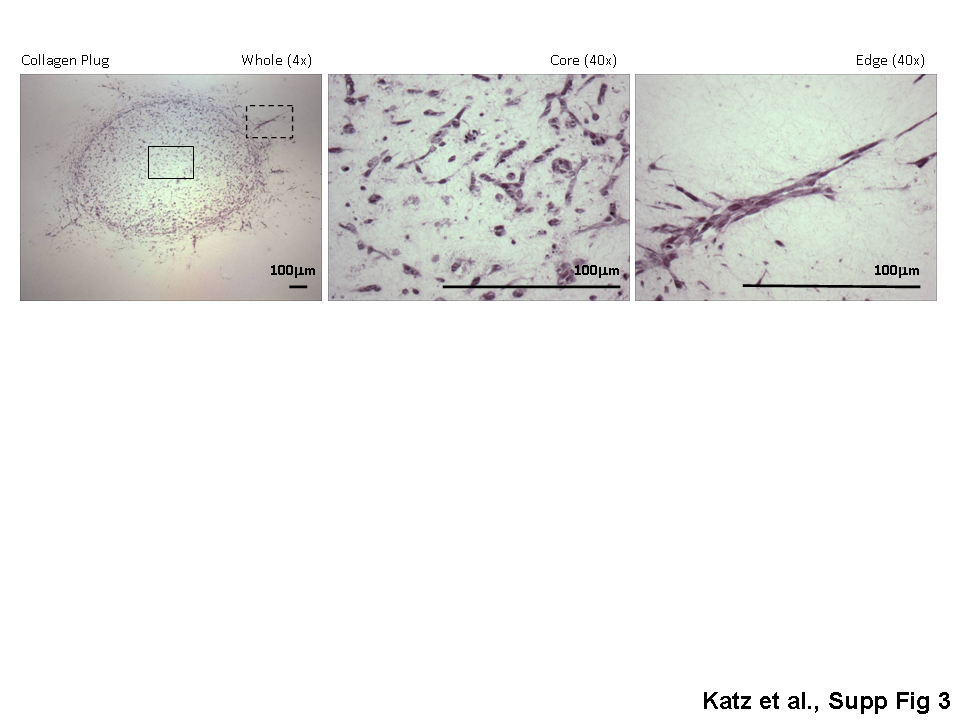

Supplement: Figure S3 — Morphology of cell-collagen assays. SUM159PT cell-collagen plugs were fixed at day 14 following a period of invasion Images of the whole plugs (4× magnification, left panel), core (middle panel) and plug edge (right panel) are shown (both 40× magnification). Note the consistently elongated cell morphology unlike cell-Matrigel assays (Figure 5a). (TIF) [file pone.0017083.s003.tif]

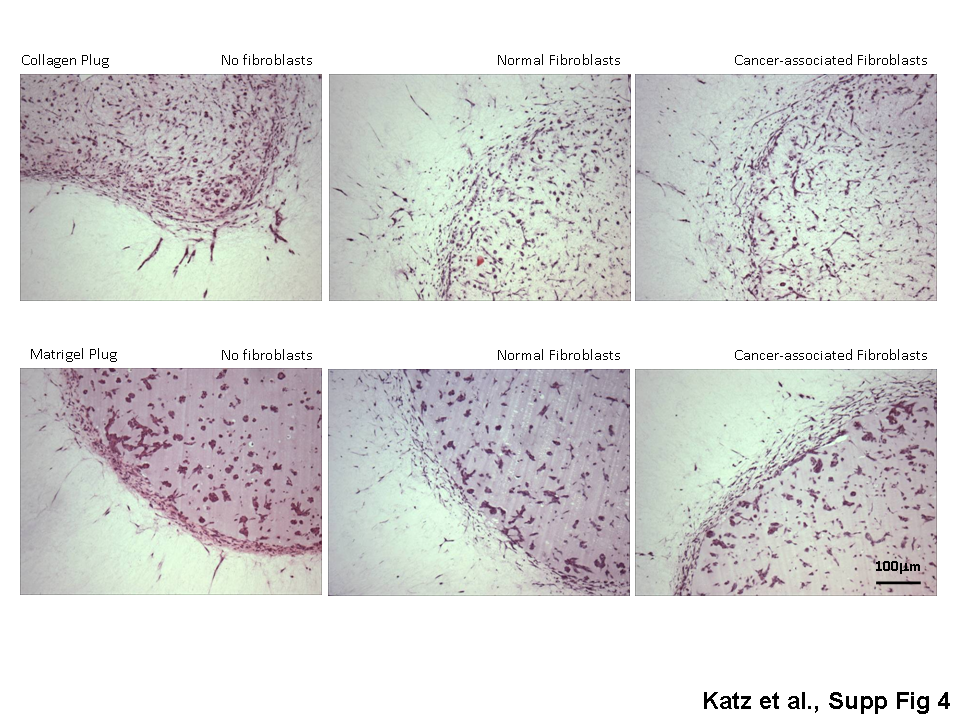

Supplement: Figure S4 — Comparable invasion of SUM159PT cells regardless of the presence or type of fibroblasts in the surrounding collagen. Comparable invasion of SUM159PT cells is seen with no, normal and cancer-associated fibroblasts. This is seen with both cell-collagen (top panel) and cell-Matrigel (bottom panel) plugs. H&E staining relating to fixation at day 6 is shown here. Bar = 100 µm. (TIF) [file pone.0017083.s004.tif]
